# A Cross-sectional Study of Knowledge, Attitude and Barriers to Colorectal Cancer Screening among Cancer Survivors

**DOI:** 10.31557/APJCP.2019.20.6.1817

**Published:** 2019

**Authors:** Yiqing Huang, Yu Yang Soon, Lay Poh Ngo, Ying Hui Dina Ee, Bee Choo Tai, Hung Chew Wong, Soo-Chin Lee

**Affiliations:** 1 *Department of Haematology-Oncology, *; 2 *Department of Radiation Oncology,*; 3 *Division of Oncology Nursing, National University Cancer Institute, *; 4 *University Medicine Cluster, *; 6 *Biostatistics Unit, Yong Loo Lin School of Medicine, National University Health System, *; 5 *Saw Swee Hock School of Public Health, *; 7 *Cancer Science Institute of Singapore, National University of Singapore, Singapore. *

**Keywords:** Survivors, colorectal cancer, screening, barriers, motivators

## Abstract

**Introduction:**

Understanding behaviour of cancer survivors is imperative as they are at risk of recurrence or second cancers. Colorectal cancer (CRC) is one of the most common cancers globally. We aim to determine the uptake rate, barriers and predictors of CRC screening among cancer survivors.

**Methods:**

Within a public hospital in Singapore, 150 non-CRC survivors were enrolled. Questionnaire on knowledge, screening behaviour, motivators and barriers towards CRC screening was administered.

**Results:**

Majority were survivors of breast (69.3%), prostate (7.3%), endometrial (4%) and ovarian (4%) cancers. More than half had high knowledge scores for CRC symptoms, screening tests and risk factors. About a third had received physician’s recommendation on CRC screening. Approximately half had undergone screening. The most common barriers to CRC screening were lack of symptoms and physician’s recommendation. Cancer survivors with higher education, higher household income, family history and those who perceived “great need” or “some need” were more likely to have undergone screening (56.4% vs 30.6%, p=0.003; 62.2% vs 41.9%, p=0.022; 70.6% vs 45.1%, p=0.048; 70.8% vs 27.4%, p<0.001). Physician’s recommendation (76.4% vs 31.6%, p<0.001) and high CRC symptom knowledge (55.8% vs 34.5%, p=0.012) were associated with increased likelihood of screening.On multivariate analysis, physician’s recommendation, higher household income and survivors’ perceived need to undergo screening remained strong predictors for CRC screening (p<0.001; p=0.010; p<0.001).

**Conclusion:**

The uptake rate of CRC screening among non-CRC survivors was modest. Physicians need to be more active in discussing CRC screening with cancer survivors as part of the survivorship care plan.

## Introduction

It was estimated that more than 60% of adults diagnosed with cancer will become long term cancer survivors, being alive for at least 5 years following their cancer diagnosis (DeSantis et al., 2014). Cancer survivors are at risk for multiple potential long term side effects of treatment which include development of a new primary cancer. The life time risk of developing a second primary cancer in cancer survivors have been estimated to be 14% (ACS, 2012). A US study involving more than two million cancer survivors reported second malignancy in 8% of patients (Donin et al., 2016). The most commonly diagnosed second malignancies were lung cancer (18%), colorectal cancer (12%) and prostate cancer (9%) respectively. In those with a second primary malignancy, more than half died from their second cancer. Thus, with the growing numbers of cancer survivors globally, it is crucial we look into their health behaviours and attitudes towards cancer screening. 

Given their long term health risks and prior cancer history, there is reason to believe cancer survivors would be more likely to undergo recommended cancer screening. However, evidence on whether this occurs is mixed. Several North American studies have reported conflicting evidence on the uptake of cancer screening in cancer survivors (Schumacher et al., 2012; Bellizzi et al., 2005; Grunfeld et al., 2012). Schumacher and colleagues (2012) found that majority of cancer survivors received recommended cancer screening and cancer survivors were more likely than people without cancer to receive cancer screening. Similarly, another large American population-based study showed that cancer survivors were more likely to undergo mammogram, Pap smear and prostate cancer screening than non-cancer controls (Bellizzi et al., 2005). However a Canadian study showed that 65% of breast cancer survivors were never screened for colorectal cancer (CRC) (Grunfeld et al., 2012). 

In Singapore, the cancer survival rates have been increasing since 1980’s (Singapore Cancer Registry, 2015). In 2010, the Ministry of Health has implemented a nation-wide guideline on CRC screening for general population where individuals aged 50 to 75 at average risk are recommended to undergo screening via annual faecal occult blood test (FOBT; guaiac FOBT or faecal immunochemical test), CT colonography every 5-yearly or colonoscopy every 10 years (Ministry of Health Singapore, 2010). In those with increased risk, such as known family history of CRC or personal history of polyps or CRC, colonoscopy screening is advised to start earlier and/or at a shorter interval. However, the uptake of CRC screening among the general population has remained low in Singapore, ranging from 20% to 27% (Wong et al., 2013; Wong et al., 2015). A report from the European Commission considered a minimum uptake of 45% in average risk population as an acceptable goal and 65% as desirable (von Karsa et al., 2013), whereas the American Cancer Society set the desirable goal at 80% (ACS, 2018). 

Currently, it is unclear in Singapore if non-CRC survivors are more likely than the general population to undergo CRC screening. Hence this study aims to determine the prevalence of CRC screening among non-colorectal cancer survivors, and identify factors that may influence the uptake of CRC screening. As the focus is to assess survivors’ attitude towards CRC screening specifically, we chose non-colorectal cancer survivors as our target population.

## Materials and Methods

*Subjects and Study Design*

Between July 2013 and September 2016, a cross-sectional study was carried out at the National University Cancer Institute, Singapore. A structured questionnaire was administered to a group of non-colorectal cancer survivors at the outpatient oncology clinic. An earlier US study reported that 43% of their cancer survivors had undergone CRC screening (Trask et al., 2005). As such, we postulated that 50% of cancer survivors would go for CRC screening, with a margin of error of 8%. Using a precision-based approach, with a 95% confidence level, a sample size of 150 was required for this study. 

Eligible patients were between 50-75 years old, with a history of non-CRC malignancy and were in cancer remission for at least 2 years. Patients with metastatic disease, on active chemotherapy or radiotherapy, with history of CRC and with pre-existing inflammatory bowel disease were excluded. Ongoing hormonal therapy for cancer was permitted. 

Participation was voluntary and all eligible patients consented to participation with a response rate of 100%. Informed consent was obtained from all participants. Four study investigators administered the questionnaire. Face-to-face interviews were conducted in a private room. Approval was obtained from the Institutional Ethics Review Board (National Health Group Domain Specific Review Board (DSRB) Reference number: 2013/00435). 

*Questionnaire*

Questionnaires were administered in English or Mandarin based on participants’ preferences. The questionnaire featured key components of the Health Belief Model (Glanz et al., 2008), including socio-demographic information, perceived health status, risk of developing CRC, knowledge of CRC symptoms, screening tests and risk factors, previous CRC screening behaviour, intent to undergo CRC screening and major motivators and barriers to CRC screening. This questionnaire was modelled after previously reported cross-sectional studies of similar nature in the Asia Pacific region (So et al., 2012; Koo et al., 2012). 

Details of cancer history, including cancer type, age at diagnosis, years of cancer remission were obtained. One’s perceived health status and perceived risk of developing CRC were assessed. To evaluate perceived susceptibility of developing CRC, survivors were asked to give a score ranging from 1 to 10, with 1 being very low likelihood, and 10 being very high likelihood. 

Knowledge of CRC symptoms, screening tests and risk factors was tested, with 1 score allocated for each correct answer. Participants were tested on their knowledge of CRC symptoms, screening tests and risk factors by answering “yes” or “no” to the following: per-rectal bleeding, fever, weight loss, change in bowel habits, burping and abdominal pain for CRC symptoms; faecal occult blood test (FOBT), blood test, abdominal X-rays and colonoscopy for CRC screening tests; and piles, smoking, family history, hypertension, age above 50 and unhealthy diet for CRC risk factors. High scores were defined as having at least 5 correct answers (out of 6) for symptoms; 3 out of 4 for screening tests; and 5 out of 6 for risk factors respectively. 

Participants were asked if they had undergone previous CRC screening, the type of tests undergone and indications for screening. They were prompted to provide reasons for not screening if they have yet to undergo screening. Survivors were also asked if they had ever received doctor’s recommendation on CRC screening. Questions to examine the need to undergo screening and future intention of undergoing screening were posed to determine whether they appreciated the rationale for screening and would take necessary future action. Finally, motivators and barriers to CRC screening were identified using a 5-point Likert scale (ranging from strongly agree to strongly disagree). Participants were asked to rate if the following were motivators to CRC screening: presence of symptoms, doctors’ recommendation, positive family history, risk of another cancer, health consciousness and media’s recommendation. In a similar fashion, they were asked to rate if the following were barriers to CRC screening: lack of symptom, lack of doctor’s recommendation, costs of test, discomfort of screening, fear of another cancer, unsure what test to screen, unsure where to undergo screening and lack of time. 

*Data Analysis*

Data was presented using appropriate descriptive statistics; categorical variables were presented as frequencies (percentages). Chi-square test was performed to identify individual risk factors that were significantly associated with actual participation in CRC screening, for example socio-demographic factors, cancer history, perceived health status and susceptibility of CRC, knowledge scores, doctor’s recommendation, motivators and barriers. Factors with significance level of p<0.1 in the univariable analysis were further considered for inclusion in the multivariable logistic regression analysis. All statistical analyses were performed using IBM SPSS Statistics Version 23, assuming a two-sided test at the 5% level of significance.

## Results

*Demographics and clinical characteristics*

One hundred and fifty non-CRC survivors were enrolled. The median age was 60 (range 50-75). Most were female (85.3%), married (77.3%) and Chinese (85.3%). More than half had received secondary level education and above (67.3%), and belonged to the lower income group, with monthly household income of less than S$5,000 (~US $3,700) (70.7%). Seventeen out of 150 survivors (11.3%) had a positive family history of CRC, of which majority of the affected family members were first degree relatives (76.5%) (Table 1).

Majority were survivors of breast cancer (69.3%), with prostate (7.3%), endometrial (4%), ovarian (4%), cervix (3.3%) and nasopharyngeal (3.3%) cancer being the other common cancers. The median age of cancer diagnosis was 54 (range 36-73). Median duration of cancer remission was 4 years (range 2-26) (Supplementary Table 1). 

More than half of the participants had a history of chronic illness (56.7%), like diabetes mellitus, hypertension, hyperlipidemia or ischemic heart disease. Majority were never-smokers (89.3%). Most participants perceived their current health status to be good or very good (62.6%). On a score of 1-10, the median perceived susceptibility score of developing CRC was 3, with only 8% of participants reporting a perceived susceptibility score of more than 5. Amongst 150 survivors, only 48% had previously undergone CRC screening. Of these, 46% (n=33) underwent FOBT, while 54% (n=39) underwent colonoscopy. Overall, about half of the participants (48%) felt that there was some need or a great need to undergo screening, and more than two-thirds (70.7%) stated that they will undergo CRC screening in the future (Supplementary Table 2).

**Figure 1:**
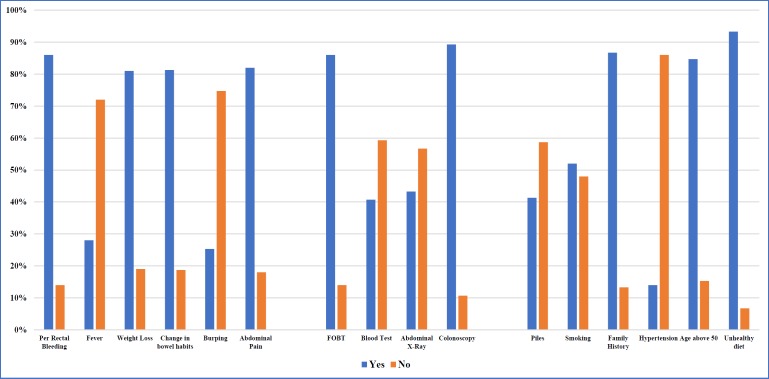
Knowledge of Symptoms, Screening Tests and Risk Factors of CRC. Participants were tested on their knowledge of CRC symptoms, screening tests and risk factors by answering “yes” or “no” to the following: per rectal bleeding, fever, weight loss, change in bowel habits, burping and abdominal pain for CRC symptoms; faecal occult blood test (FOBT), blood test, abdominal X-rays and colonoscopy for CRC screening tests; and piles, smoking, family history, hypertension, age above 50 and unhealthy diet for CRC risk factors. One score was allocated for each correct answer. A high score for CRC symptom was defined as having at least 5 correct answers (out of 6); medium score as having 3-4 correct answers; and low score as having 0-2 correct answers. A high score for CRC screening test was defined as having at least 3 correct answers (out of 4); medium score as having 2 correct answers; and low score as having 0-1 correct answer. A high score for CRC risk factor was defined as having at least 5 correct answers (out of 6); medium score as having 3-4 correct answers; and low score as having 0-2 correct answers

*Knowledge of Colorectal Cancer: Symptoms, Screening and Risk Factors (* Figure 1 *)*

Overall, 63.3%, 61.3% and 55.3% of survivors respectively obtained high knowledge scores for CRC symptoms, screening tests and risk factors (Table 2), while 34%, 32% and 22% had perfect scores for the respective categories. More than 80% of survivors knew the symptoms of CRC: rectal bleeding, weight loss, change in bowel habits and abdominal pain. The majority was aware that FOBT and colonoscopy are appropriate CRC screening tools (86%, 89.3% respectively). However, as high as 40% of survivors mistook blood test and abdominal X-rays as appropriate screening tests.

Most participants (more than 80%) acknowledged positive family history, age more than 50 and unhealthy diet (defined as diet high in fat and low in fiber) as risk factors for developing CRC. Only 52% was aware that smoking can increase the risk of CRC. Forty-one percent (41.3%) of survivors mistook piles as a risk factor.

Survivors with higher education (secondary school and above) had higher knowledge for CRC symptoms compared to those of lower education (73.3% vs 42.9%, p<0.001), whereas factors like age, race and household income did not correlate with higher CRC symptom knowledge. 

*Colorectal Cancer Screening Behaviour*

About half (48%) of the 150 non-CRC survivors had previously undergone CRC screening at the time of the survey. Despite regular follow-ups with their oncologists, only 36.7% of survivors ever received doctor’s recommendation to undergo CRC screening (Supplementary Table 2).

Amongst survivors who had undergone screening, the most commonly cited reasons for screening include part of routine screening (41.6%), presence of symptoms (31.9%), doctor’s recommendation (23.6%) and family history (2.7%). Lack of symptoms (73.1%), and lack of doctor’s recommendation (14.1%) were the most commonly cited reasons against screening. Other reasons cited include fear of another cancer (5.1%), lack of awareness for the need for screening (3.8%), fear of pain and discomfort from screening (2.6%) and that screening was troublesome (1.3%) (Supplementary Table 2). 

*Factors influencing CRC screening behaviour (* Table 1 *,* 2 *)*

Cancer survivors who are more highly educated (secondary school and above), have higher household income, and those with family history were more likely to have undergone CRC screening compared to those of lower education, lower income and those without family history, respectively (56.4% vs 30.6%, p=0.003; 62.2% vs 41.9%, p=0.022; 70.6% vs 45.1%, p=0.048). Survivors who are single are less likely to undergo CRC screening than non-single survivors (OR 0.313, 95% CI 0.108 to 0.913). Survivors who perceived “some need” or “great need” to undergo screening were more likely to do so compared to those who perceived “little need” or “no need” (p<0.001). 

Physician’s recommendation (76.4% vs 31.6%, p<0.001) and high CRC symptom knowledge (55.8% vs 34.5%, p=0.012) were also associated with increased likelihood of screening. Survivors with high CRC screening test knowledge were more likely to undergo screening, though this did not reach statistical significance (p=0.05)

On multivariate analysis, physician’s recommendation, higher household income and survivors’ perceived need to undergo screening remained significantly correlated with actual CRC screening (OR 7.15 (95% CI 3.00 – 17.1), p<0.001; OR 3.32 (95% CI 1.33- 8.31), p=0.01; OR 7.10 (95% CI 3.08-16.4), p<0.001), whereas education level, family history and high CRC symptom knowledge were no longer significant (Table 3). 

**Figure 2:**
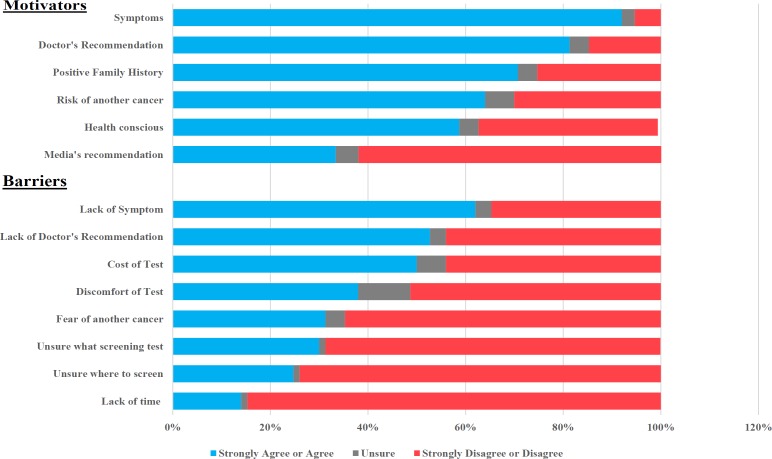
Motivators and Barriers to CRC Screening. Using a 5-point Likert scale (ranging from strongly agree to strongly disagree), participants were asked to rate if the following were motivators to CRC screening: presence of symptoms, doctors’ recommendation, positive family history, risk of another cancer, health consciousness and media’s recommendation. In a similar fashion, participants were asked to rate if the following were barriers to CRC screening: lack of symptom, lack of doctor’s recommendation, costs of test, discomfort of screening, fear of another cancer, unsure what test to screen, unsure where to undergo screening and lack of time

*Motivators and Barriers Influencing CRC Screening (* Figure 2 *)*

The most commonly cited motivators (strongly agree or agree) were presence of symptoms (92%), physician’s recommendation (81.4%) and family history (70.7%). On the other hand, the most commonly cited barriers (strongly agree or agree) include lack of symptom (62%), lack of physician’s recommendation (52.7%), costs of screening tests (50%) and discomfort of screening tests (38%). About one-third cited fear of another cancer and lack of knowledge of what screening tests to be performed as barriers (31.3%; 30% respectively). 

Survivors who cited “physician’s recommendation”, “health-consciousness” and “risk of another cancer” as motivators for screening were more likely to have undergone CRC screening compared to their counterparts, respectively (52.5% vs 22.7%, p=0.01; 65.9% vs 25%, p<0.001; 56.3% vs 31.1%, p=0.005). However, those who cited “symptoms” and “positive family history” as motivators were not more likely to have undergone CRC screening than those who did not cite these as motivators (47.8% vs 62.5%, p=0.419; 50% vs 47.4%, p=0.78) (Supplementary Table 3).

On the other hand, participants who cited “lack of doctor’s recommendation”, “lack of symptom”, “fear of getting another cancer”, “discomfort of screening”, “costs”, “unsure what test to be done” and “unsure where to perform screening” as barriers were all less likely to have undergone CRC screening than their counterparts (all p<0.01) (Supplementary Table 4).

**Table 1:** Factors Influencing CRC Screening Behaviour (Demographic Variables)

	Ever Gone for Screening		
	Yes	No	P value
Sociodemographic Factors			
Gender			
Male (n=22)	12 (54.5%)	10 (45.5%)	0.506
Female (n=128)	60 (46.9%)	68 (53.1%)	
Age (years)			
Less than 55 (n=43)	19 (44.2%)	24 (55.8%)	
55-64 (n=60)	28 (46.7%)	32 (53.3%)	0.67
65 and above (n=47)	25 (53.2%)	22 (46.8%)	
Race			
Chinese (n=128)	62 (48.4%)	66 (51.6%)	
Malay (n=16)	6 (37.5%)	10 (62.5%)	0.67
Indian (n=3)	2 (66.7%)	1 (33.3%)	
Others (n=3)	2 (66.7%)	1 (33.3%)	
Education			
Primary school and below (n=49)	15 (30.6%)	34 (69.4%)	0.003
Secondary school and above (n=101)	57 (56.4%)	44 (43.6%)	
Marital Status			
Single (n=20)	5 (25%)	15 (75%)	<0.001
Married (n=116)	54 (46.6%)	62 (53.4%)	
Widowed or Divorced (n=14)	13 (92.9%)	1 (7.1%)	
Work Status			
Full time or Part time (n=75)	36 (48.0%)	39 (52.0%)	0.97
Homemaker (n=49)	24 (49%)	25 (51%)	
Unemployed or Retired (n=26)	12 (46.2%)	14 (53.8%)	
Income			
Less than S$5000 (~US$3700) per month (n=105)	44 (41.9%)	61 (58.1%)	0.022
S$5000 (~US$3700) per month and above (n=45)	28 (62.2%)	17 (37.8%)	
Family history of CRC			
Yes (n=17)	12 (70.6%)	5 (29.4%)	0.048
No (n=133)	60 (45.1%)	73 (54.9%)	

**Table 2:** Factors Influencing CRC Screening Behaviour (Other Variables)

	Ever Gone for Screening		
	Yes	No	P value
Cancer History			
Breast Cancer (n=104)	50 (48.1%)	54 (51.9%)	0.977
Other Cancers (n=46)	22 (47.8%)	24 (52.2%)	
Years of cancer remission			
Less than 5 (n=76)	38 (50%)	38 (50%)	0.619
5 or more (n=74)	34 (45.9%)	40 (54.1%)	
Age of cancer diagnosis (years)			
Less than 50 (n=46)	19 (41.3%)	27 (58.7%)	0.275
50 and above (n=104)	53 (51.0%)	51 (49.0%)	
Perceived health status			
Fair or Poor (n=56)	26 (46.4%)	30 (53.6%)	0.766
Good or Very Good (n=94)	46 (48.9%)	48 (51.1%)	
Perceived susceptibility to CRC			
5 or less (n=138)	65 (47.1%)	73 (52.9%)	0.455
More than 5 (n=12)	7 (58.3%)	5 (41.7%)	
Perceived Need to undergo screening			
Little need or no need (n=73)	20 (27.4%)	53 (72.6%)	<0.001
Some need or great need (n=72)	51 (70.8%)	21 (29.2%)	
Knowledge Score on CRC Symptoms			
High Score (n=95)	53 (55.8%)	42 (44.2%)	0.012
Low or Middle Scores (n=55)	19 (34.5%)	36 (65.5%)	
Knowledge Score on CRC Screening tests			
High Score (n=92)	50 (54.3%)	42 (45.7%)	0.05
Low or Middle Scores (n=58)	22 (37.9%)	36 (62.1%)	
Knowledge Score on CRC Risk factors			
High Score (n=83)	38 (45.8%)	45 (54.2%)	0.545
Low or Middle Scores (n=67)	34 (50.7%)	33 (49.3%)	
Doctors’ Recommendation			
Yes (n=55)	42 (76.4%)	13 (23.6%)	<0.001
No (n=95)	30 (31.6%)	65 (68.4%)	

**Table 3:** Multi-Variable Analysis: Factors affecting CRC Screening

Multivariate Analysis: Factors affecting CRC screening			
Factors associated with CRC screening	Odds Ratio	95% CI	p-value
Household Income	3.32	1.33 – 8.31	0.01
Doctor’s Recommendation	7.15	3.00 - 17.7	<0.001
Perceived Need to Undergo Screening	7.1	3.08 - 16.4	<0.001

95% CI, 95% confidence interval; CRC, colorectal cancer

## Discussion

There is substantial interest globally in the health care needs of cancer survivors. Their growing numbers reinforce the need to explore their health behaviors and attitudes towards cancer screening. In our study, only about half of cancer survivors have undergone CRC screening. Although this figure is double that of the screening uptake rate (~25%) reported amongst our general population (Wong et al., 2013; Wong et al., 2015), it can be improved. The utilization rates of CRC screening among non-CRC survivors in Singapore are fairly similar to other countries. A Korean study reported that less than 30% of lung cancer survivors adhered to colorectal screening recommendation (Park et al., 2017). Trask and colleagues (2005) reported a uptake CRC screening rate of 43% among US cancer survivors. These highlight that CRC screening practices among cancer survivors are less than optimal globally. 

Despite paying regular visits to their oncologists, only one-third of cancer survivors in our study ever received doctors’ recommendation to undergo CRC screening. Doctors’ recommendation is a strong motivator for survivors to undergo screening: it is the second most commonly cited motivator in our study, and those who actually received doctors’ recommendation were more likely to undergo screening in this cohort. This result is consistent with another study conducted amongst the Singapore general population and a Korean study conducted amongst lung cancer survivors (Wong et al., 2013; Park et al., 2017). Physicians are the most influential vehicle for promoting behavioural change in cancer screening (Mandelblatt and Kanetsky, 1995; Aziz and Rowland, 2003), and should realize their advice serve as significant impetus for patients to undergo screening. 

While more time can be spent to counsel survivors on health behaviours, high patient load and limited clinic time per patient are often challenges faced in the real world. A recent STEP study evaluating oncologists’ perspectives of cancer survivorship care showed that lack of time and evidence based guidelines were the main barriers to optimal survivorship care in Asia (Chan et al., 2017). Thus, strategies are needed to overcome these barriers. Lately, a communication strategy named “teachable moments” has shown promise as an approach to discuss behaviour change with patients (Flocke et al., 2014). “Teachable moments” are “cueing events” or naturally occurring health events that lead individuals to make health behaviour changes (Cohen et al., 2011). 

We identified that cancer survivors with higher education, higher income, positive family history and higher CRC symptom knowledge were more likely to undergo CRC screening. Higher income remained significantly correlated with CRC screening after multivariate analysis. Income and education are known major contributors to inequalities in cancer screening practices. A Korean study showed that participants of higher income and education were more likely to undergo colorectal and gastric cancer screening (Kim and Hwang, 2016). Similar findings were reported for mammogram screening in Singapore (Teo et al., 2013) and CRC screening in the United Kingdom (Solmi et al., 2015). Despite government subsidies for FOBT and colonoscopy in Singapore, awareness and uptake for CRC screening remains low (Wong et al., 2013; Singapore National Health Survey, 2010). A possible explanation is that cancer screening participation may be influenced by other factors correlated to income (Sabates and Feinstein, 2006; Wardle et al., 2004; Stimpson et al., 2012; Finney et al., 2004). For example, people of lower income with unpaid sick leave may have more difficulties taking time off work to seek preventive care services (Peipins et al., 2012; Kim et al., 2015). They may also have a lower health literacy level, which can exert a profound influence on their decision making about screening (Rakowski et al., 2006; Bao et al., 2007).

In our study, there was high knowledge of CRC amongst cancer survivors. More than 80% knew the symptoms, screening tests and risk factors for CRC. However, despite the high knowledge and a personal history of cancer, close to half believed there is ‘little need’ or ‘no need’ for CRC screening. This phenomenon is worrisome because survivors may have the knowledge about the disease, but lack motivation to take active steps to undergo screening. This is similar to an earlier Korean study that found that while an educational intervention could improve the knowledge of second primary cancer among cancer survivors, it did not improve their screening uptake (Shin et al., 2012). 

The main barriers to CRC screening identified in this study were lack of symptom (62%), lack of doctor’s recommendation (52.7%) and costs of screening tests (50%). These barriers are similar to those cited amongst the general population (Honein-AbouHaidar et al., 2016). They stem largely from the lack of awareness of CRC screening and the misperception that screening is only necessary when symptoms develop. It is thus prudent to educate our patients on the importance of screening despite the absence of symptoms. We believe cost of screening may be more of a misperception than a real barrier. In Singapore, FOBT is priced at S$30 (~US $22) and lower income Singaporeans can gain free testing under the Integrated Screening Program. The estimated cost of a colonoscopy screening is approximately S$250 and S$1,000 for subsidized and non-subsidized patients respectively in a public institution. Since June 2011, the government has approved the extension of Medisave for colonoscopy examination to make it more affordable for Singaporeans. Medisave is a national medical savings scheme that mandates Singaporeans to put aside part of their income into a Medisave account to meet hospitalization, surgery and outpatient expenses. Singaporeans can now claim up to S$950 from their Medisave for colonoscopy screening. Patients should be educated properly on the costs of the procedures and subsidies that are available to them. 

Our study had a high number of breast cancer survivors (69.3%). CRC is a known common second malignancy amongst breast cancer survivors (Donin et al., 2016), but whether breast cancer survivors are at increased risk of developing CRC remains debatable. A study in Sweden involving close to 180,000 breast cancer patients reported a 1.6-fold increased risk of CRC compared to the general population (Lu et al., 2016). On the other hand, a recent meta-analysis involving more than one million breast cancer survivors showed the incidence of CRC to be similar to controls. However, those diagnosed with breast cancer below the age of 50 had a 2.5-fold increased risk of CRC compared to their older counterparts (Lai et al., 2017).

Several limitations are acknowledged. Firstly, response bias is a recognized limitation of face-to-face interviews, where pressure to provide a socially acceptable answer may result in over-reporting of positive health behaviour (Furnham, 1986). Secondly, there is recall bias as participation of screening tests and physician recommendation recalls were self-reported, and not verified by medical records. Thirdly, there was an uneven distribution of gender in our study, with significantly more female compared to male survivors. This is largely contributed by the large numbers of breast cancer survivors in this study. Fourthly, our questionnaire did not specifically identify screening intervals among our cancer survivors, which would have been useful to determine screening compliance, especially when frequency intervals of each screening tool differ. Lastly, the use of a structured questionnaire with set response options also limit the outcomes that can be reached within a survey analysis. Nonetheless, we have designed the questionnaire using key components of the Health Belief Model to predict behaviours of the cancer survivors. To our knowledge, this is one of few studies in Asia looking specifically at attitudes and barriers of CRC screening amongst a group of non-CRC cancer survivors. Notably, there is no missing data in our study, suggesting strength and accuracy in our results reported.

In conclusion, the uptake rate of CRC screening among non-CRC survivors in Singapore was modest. Doctor’s recommendation is a strong driving force for patients to undergo screening. 

This study highlights the importance of educating and promoting cancer screening among our cancer survivors. Going forward, oncologists should take active interest in promoting cancer screening, and cancer institutions can set up cancer screening programs specifically tailored for cancer survivors. 

*List of abbreviations:*

CRC: Colorectal Cancer; FOBT: Faecal occult blood test; CT: Computed tomography; ASRS: Aged-standardised relative survival 
